# Zika virus disrupts gene expression in human myoblasts and myotubes: Relationship with susceptibility to infection

**DOI:** 10.1371/journal.pntd.0010166

**Published:** 2022-02-16

**Authors:** Ingo Riederer, Daniella Arêas Mendes-da-Cruz, Guilherme Cordenonsi da Fonseca, Mariela Natacha González, Otavio Brustolini, Cássia Rocha, Guilherme Loss, Joseane Biso de Carvalho, Mariane Talon Menezes, Lidiane Menezes Souza Raphael, Alexandra Gerber, Myrna Cristina Bonaldo, Gillian Butler-Browne, Vincent Mouly, Vinicius Cotta-de-Almeida, Wilson Savino, Ana Tereza Ribeiro de Vasconcelos

**Affiliations:** 1 Laboratory on Thymus Research, Oswaldo Cruz Institute, Oswaldo Cruz Foundation, Rio de Janeiro, Brazil; 2 National Institute of Science and Technology on Neuroimmunomodulation (INCT-NIM); Oswaldo Cruz Institute, Oswaldo Cruz Foundation, Rio de Janeiro, Brazil; 3 Rio de Janeiro Research Network on Neuroinflammation (RENEURIN), Oswaldo Cruz Institute, Oswaldo Cruz Foundation, Rio de Janeiro, Brazil; 4 School of Pharmacy and Biomedical Sciences, University of Central Lancashire, Preston, England, United Kingdom; 5 Bioinformatics Laboratory, National Laboratory for Scientific Computing, Petropolis, Rio de Janeiro, Brazil; 6 Department of Genetics, Institute of Biology, Federal University of Rio de Janeiro, Rio de Janeiro, Brazil; 7 Laboratory of Molecular Biology of Flavivirus, Oswaldo Cruz Institute, Oswaldo Cruz Foundation, Rio de Janeiro, Brazil; 8 Sorbonne Université, Inserm, Institut de Myologie, Centre de Recherche en Myologie, Paris, France; INSERM, FRANCE

## Abstract

The tropism of Zika virus (ZIKV) has been described in the nervous system, blood, placenta, thymus, and skeletal muscle. We investigated the mechanisms of skeletal muscle susceptibility to ZIKV using an *in vitro* model of human skeletal muscle myogenesis, in which myoblasts differentiate into myotubes. Myoblasts were permissive to ZIKV infection, generating productive viral particles, while myotubes controlled ZIKV replication. To investigate the underlying mechanisms, we used gene expression profiling. First, we assessed gene changes in myotubes compared with myoblasts in the model without infection. As expected, we observed an increase in genes and pathways related to the contractile muscle system in the myotubes, a reduction in processes linked to proliferation, migration and cytokine production, among others, confirming the myogenic capacity of our system in vitro. A comparison between non-infected and infected myoblasts revealed more than 500 differentially expressed genes (DEGs). In contrast, infected myotubes showed almost 2,000 DEGs, among which we detected genes and pathways highly or exclusively expressed in myotubes, including those related to antiviral and innate immune responses. Such gene modulation could explain our findings showing that ZIKV also invades myotubes but does not replicate in these differentiated cells. In conclusion, we showed that ZIKV largely (but differentially) disrupts gene expression in human myoblasts and myotubes. Identifying genes involved in myotube resistance can shed light on potential antiviral mechanisms against ZIKV infection.

## Author summary

The ZIKV was responsible for a large pandemic surge between 2015 and 2016; Brazil was the epicenter with very high numbers of congenital ZIKV syndrome that included microcephaly, myalgia, among other manifestations. We have observed in previous work that muscle progenitor cells (myoblasts) are infected by ZIKV, while differentiated cells (myotubes) do not present viral proteins. In the present study, we confirmed human myoblasts susceptibility to ZIKV infection and myotubes resistance. In fact, we show that myotubes are also susceptible to ZIKV entry but control the productive infection. We also elucidated the transcriptional mechanisms triggered by the ZIKV, which stimulated a more complex intrinsic antiviral response in myotubes than in myoblasts. Such particular gene modulation of the differentiated cells could offer clues to antiviral mechanisms. It would explain susceptibility differences between adults, where muscle progenitor cells are quiescent in the steady-state, and foetuses that have large amounts of these cells proliferating in order to grow muscles.

## Introduction

The ZIKV is an arthropod-borne virus (arbovirus) from the *Flaviviridae* family and genus Flavivirus, which also includes dengue, yellow fever, and West Nile viruses. Congenital ZIKV infection results in a range of neurological abnormalities in children, collectively defined as congenital Zika syndrome [1,2], comprising, among others, microcephaly, decreased brain tissue, absence of brain structures, eye abnormalities, as well as congenital contractures such as arthrogryposis [3].

Several studies have described the tropism of ZIKV for different cell types of the central nervous system like microglia, neural stem cells, and progenitor cells, as well as in other tissues such as skin, blood, placenta, retina, thymus, as well as smooth and skeletal muscle [4–10].

A spectrum of neurological outcomes was described in toddlers and preschoolers that have been congenitally infected by the ZIKV [11]. In this study, children were separated into groups termed corticospinal or neuromuscular based on their clinical signs, with or without dyskinetic findings. Comorbidities in the neuromuscular group were highly prevalent and had worse functional outcomes than the corticospinal group [11]. Furthermore, infants can develop features consistent with fetal immobility, ranging from dimples, distal hand/finger contractures and foot mispositioning to generalized arthrogryposis [12].

Under normal conditions, the adult skeletal muscle in mammals is a very stable tissue but has a remarkable ability to repair after injury due to the presence of adult stem cells called satellite cells.

The pool of satellite cells can provide myogenic cells, which proliferate (myoblasts), differentiate and fuse into myotubes, leading to the formation of new multinucleated myofibers, physiologically formed by the fusion of many myoblasts during embryonic and fetal development [13].

Satellite cells are quiescent cells located in niches on the surface of myofibers, under the basement membrane [14,15]. In adults, they can be activated and recruited to generate myonuclei for muscle fiber homeostasis or for the more sporadic demands such as myofiber hypertrophy or repair [16]. Importantly, in addition to producing the progeny necessary for differentiation, satellite cells also maintain their own population by self-renewing [17].

Previous data revealed that myoblasts are permissive to arboviruses, including the chikungunya virus (CHIKV) [18]. In the same vein, it has been shown that primary disorders caused by ZIKV, such as microcephaly and arthrogryposis [2,3], occur during the fetal phase and infancy when myoblasts are continually merging into myotubes to promote muscle growth.

Recently, it was demonstrated *in vitro* that human myoblasts could be infected by ZIKV, whereas myotubes are resistant to infection [10]. These findings indicate that the susceptibility to infection is dependent on the differentiation state of the muscle cells. Such a susceptibility was observed with three different virus isolates and with primary myoblasts from three different donors, as well as with a muscle cell line. In all cases, ZIKV infection of myoblasts was productive, indicating that muscle cells can account for a peripheral site of viral amplification, which might contribute to the pathophysiology of ZIKV infection.

However, a series of questions related to the differential responses of myoblasts and myofibers to ZIKV infection remained unanswered. The corresponding changes in transcription control could shed light on the mechanisms leading from a permissive state in myoblasts to a resistant state in myotubes. Herein we analyzed functional and gene expression profiles (including transcriptome and miRNome) in ZIKV infected human myoblasts and myotubes compared to non-infected controls, aiming to provide clues to the mechanisms involved in susceptibility *versus* the resistance of skeletal muscle cells to ZIKV infection.

## Results

### Characterization of ZIKV infection of human skeletal muscle cells

**Fig 1:**
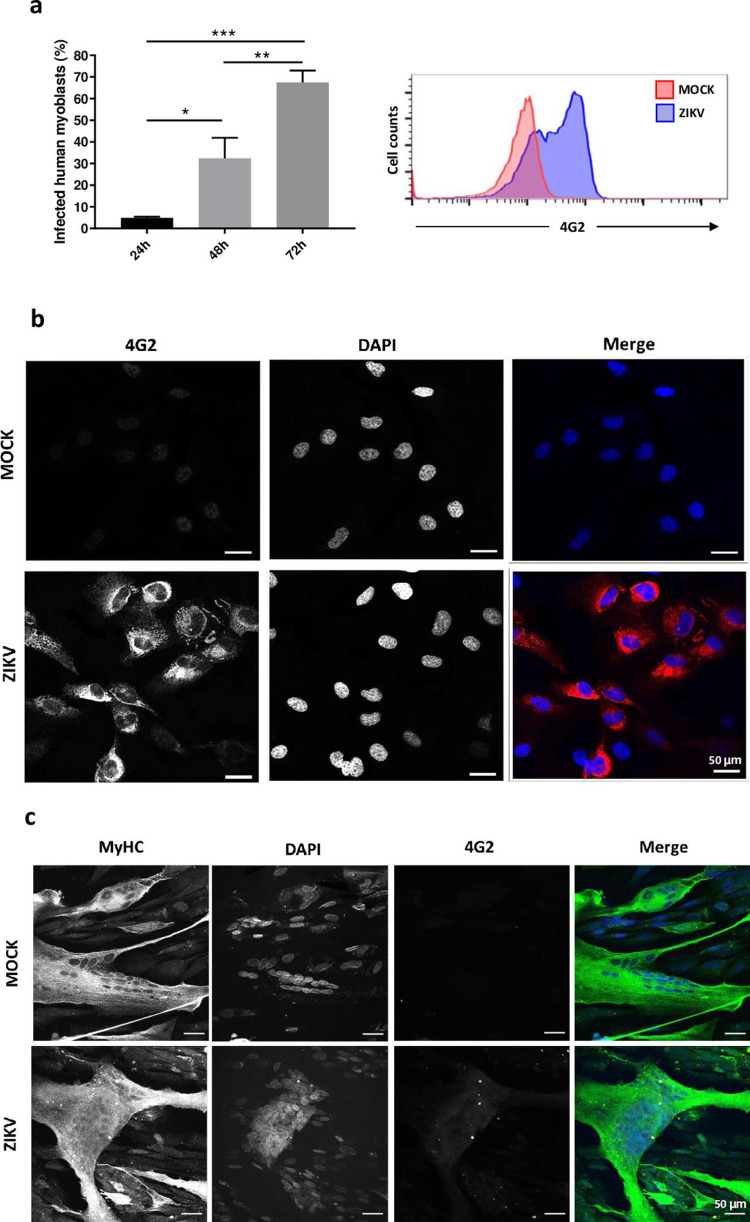
Human cultured myoblasts (but not myotubes) can be infected by Zika virus and generate intracellular viral particles. (**A**) The LHCN-M2 myoblast cell line was infected with ZIKV (MOI = 0.1), and the percentage of 4G2^+^ cells was detected 24- (n = 2), 48- (n = 2) and 72- (n = 4) hours post-infection by flow cytometry. Each time point is represented by mean ± standard error. Results were analysed by one-way ANOVA and Tukey post-test. *p < 0.05, **p < 0.01, ***p < 0.001. (**B**) Representative microscopic fields of mock *versus* ZIKV myoblast cultures immunostained with the 4G2 anti-flavivirus antibody at 72 hours post-infection, showing immunostainings for 4G2 (in red) and DAPI (nuclei, in blue). Panel **c** depicts LHCN-M2 cells differentiated into myotubes (herein revealed by the myosin heavy chain—MyHC—expression, shown in green (with nuclei stained in blue with DAPI). In these cultures, we could not detect ZIKV proteins with the 4G2 antibody (n = 3).

To investigate the mechanisms involved in skeletal muscle susceptibility or resistance to ZIKV infection, we applied an *in vitro* model of human skeletal muscle myogenesis, in which proliferating myoblasts can be differentiated in myotubes (see methods). For this, we used the immortalized human cell line LHCN-M2 [19], being positive for desmin and CD56, myogenic markers (S1 Fig) and can be induced to differentiate to form multinucleated myotubes expressing myosin heavy chain (MyHC), a sarcomeric protein expressed only in differentiated muscle cells (Fig 1C). Using 0.1 MOI dose of infection, we observed that human myoblasts are susceptible to the RIO-U1 ZIKV infection, whereas myotubes are resistant. Around 70% of myoblasts were positive for the presence of 4G2-labeled viral envelope protein, 72 hours post-infection (Fig 1A and 1B), whereas myotubes (positive for MyHC labeling) remained negative for 4G2 immunostaining at the same time point (Fig 1C).

### Differential gene expression during myoblast differentiation

Considering the different susceptibility of myoblasts to ZIKV infection compared to myotubes, we first analyzed gene expression in cultures of myoblasts *versus* myotubes in the absence of any infection. We found that 2,200 differentially expressed genes (DEGs) were significantly upregulated in myotubes, as compared to myoblasts, whereas 2,364 genes were downregulated, comprising various RNA classes (miRNAs not being included herein), including protein-coding and long non-coding RNAs (Tables 1 and S1A and S1B).

**Table 1:** Summary of the differentially Expressed Genes and miRNAs identified by RNAseq and small RNAseq in human myoblasts and myotubes, infected or not by ZIKV.

RNA type	Expressed Genes	Upregulated*	Downregulated*	DEGs*
**Normal myotubes over normal myoblasts**				
Protein Coding	16332	1524	2193	3717
Long Non-Coding RNAs**	6327	427	106	533
other RNA Classes***	8081	249	65	314
Total genes	30740	2200	2364	4564
miRNAs	1522	96	80	176
**ZIKV-infected myoblasts over normal myoblasts**				
Protein Coding	16808	434	178	612
Long Non-Coding RNAs**	6668	42	13	55
other RNA Classes***	8476	31	15	46
Total genes	32222	507	206	713
miRNAs	1531	13	19	32
**ZIKV-induced myotubes over normal myotubes**				
Protein Coding	16430	1260	417	1677
Long Non-Coding RNAs**	6496	77	112	189
other RNA Classes***	8130	53	58	111
Total genes	31056	1390	587	1977
miRNAs	1574	6	4	10

*Statistical significance of DESEq2 with adjusted p-value < 0.05 and Log2 Fold Change > |1.0|

**Antisense, lincRNA and bidirectional promoter lncRNA were included in this category

*** All other RNA classes not included in the categories as mentioned above.

**Fig 2:**
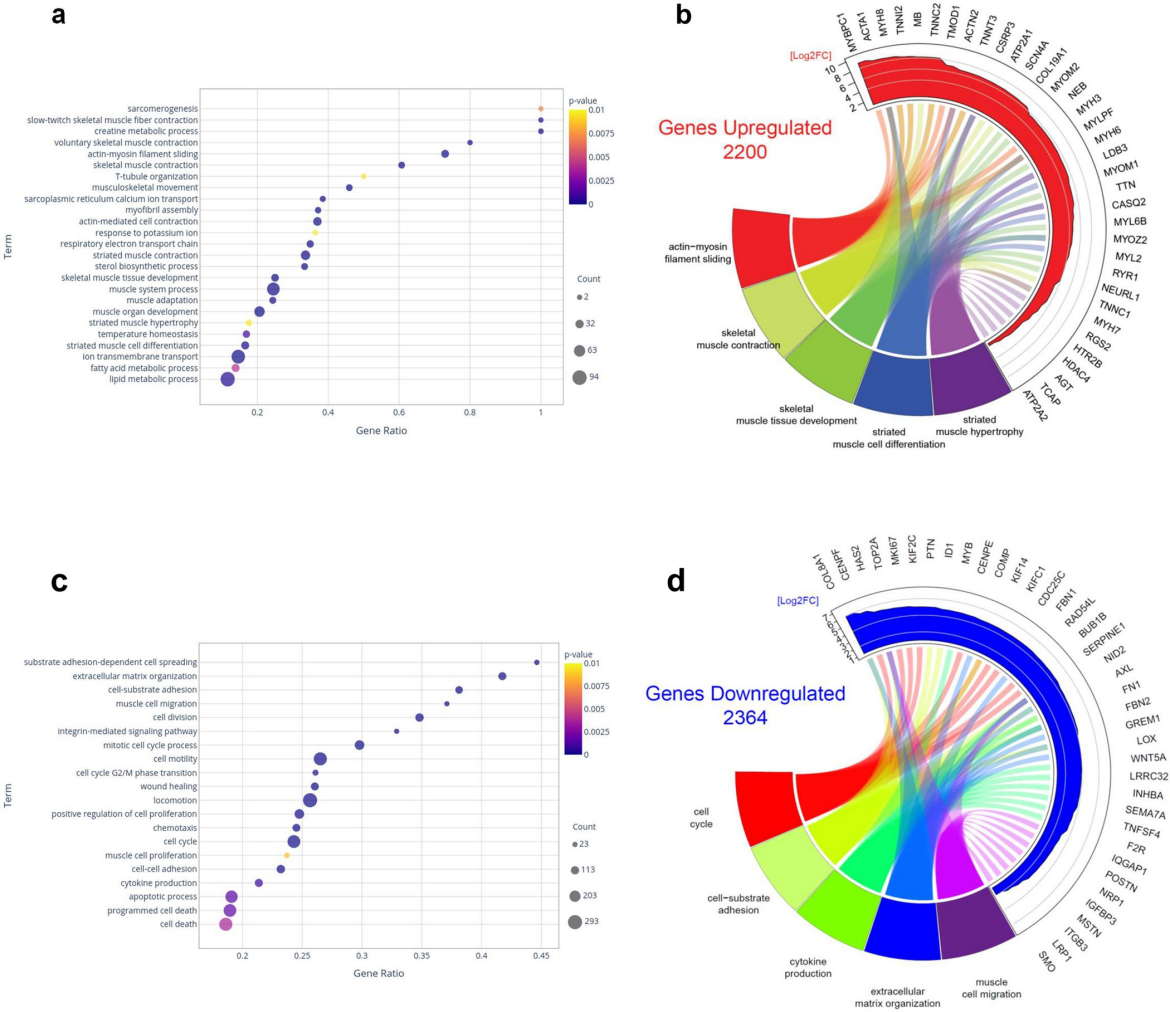
*In vitro* human myoblast differentiation into myotubes induces multiple alterations in gene expression. (**A**) Dot plot illustrating the GO biological process annotations upregulated in myotubes over myoblasts reflecting myoblast differentiation. (**B**) Circos plot representing the total numbers of genes upregulated in myotubes over myoblasts and the 10 most highly upregulated genes among 5 selected GO biological processes. (**C**) Dot plot illustrating the GO biological process annotations downregulated in myotubes over myoblasts. (**D**) Circos plot representing the total numbers of genes downregulated in myotubes over myoblasts and the 10 most highly downregulated genes among 5 selected GO biological processes. The whole list of differentially expressed genes between myotubes and myoblasts can be seen in S1 Table.

Interestingly, Volcano plot analysis revealed that, although more genes were downregulated in myotubes, these cells showed a higher number of upregulated genes with 10-fold enhanced expression (S2 Fig). As expected, when we clustered the upregulated protein-coding genes by means of Gene Ontology (GO), myotube-derived upregulated pathways were related to skeletal muscle cell development, differentiation, contraction, ion transport and muscle metabolism (Fig 2A). Of note, the myotubes used in this study were analyzed at 6 days of differentiation.

To highlight the most upregulated genes, we chose 5 relevant upregulated GOs and identified the 10 most upregulated genes within each of those GO pathways (Fig 2B). Most of these genes, including those with more than 8-fold log changes, are involved in the differentiation and contraction of the striated skeletal muscle. By contrast, differentiation of myotubes from myoblasts led to the gene downregulation in GOs involved in cell proliferation/cell cycle, cell migration, cytokine production, adhesion, extracellular matrix organization and cell death/apoptosis (Fig 2C). Fig 2D shows the most downregulated genes in 5 selected GOs. Some with approximately 6 log fold changes, these genes are related to cell cycle and migration and are expected to be blocked or reduced in the transition of myoblasts to myotubes.

Differences in gene expression between myoblasts and myotubes also concerned miRNAs, with 96 miRNAs being upregulated and 80 downregulated in myotubes, as compared to myoblasts (Table 1). In a similar way to the expression of the mRNAs, but with a reverse pattern, the upregulated miRNAs also presented the highest fold change values, with the miR-499a-5p being the most upregulated with a log2 fold change of 8.446, and the miR-3923-3p the most downregulated with a log2 fold change of -6.518 (S2A Table). Interestingly, the miR-499a-5p is a skeletal muscle-specific microRNA related to inhibiting both cell migration and proliferation [20].

We also searched for the validated miRNA targets that present the inverse expression pattern from the miRNAs. This miRNA-target network revealed 1,468 up- and downregulated genes (S2B Table). The GO enrichment analysis of these target genes showed that they were mostly related to DNA replication and cell division (S3 Fig).

### ZIKV disrupts gene expression profiles in both myoblasts and myotubes

Since myoblasts, but not myotubes, are permissive to intracellular ZIKV replication, we next searched to evaluate gene expression profiles that could provide clues to explain this difference. We found that 507 differentially expressed genes were significantly upregulated in ZIKV-infected myoblasts and 1,390 in myotubes that were in contact with ZIKV (ZIKV-treated myotubes), as compared to the corresponding non-infected (mock) cells. Conversely, 206 genes were downregulated in infected myoblasts and 587 in ZIKV-treated myotubes, including various RNA classes, as seen in Tables 1 and S1C and S1D.

The corresponding Venn diagram revealed that among the 507 upregulated genes in infected myoblasts, 220 were also upregulated in infected myotubes, but 4 were downregulated. Additionally, among the 206 downregulated genes in myoblasts, 35 were also downregulated in myotubes, whereas 13 were upregulated. In contrast, myotubes upregulated 1,390 genes after ZIKV-incubation, with 220 being also upregulated in myoblasts, whereas 13 were downregulated. Furthermore, 35 out of the 587 genes downregulated in ZIKV-treated myotubes were also downregulated in myoblasts, whereas 4 were upregulated. These findings are summarized in S4 Fig.

Volcano plot analysis revealed that not only there were more genes upregulated in both myoblasts and myotubes exposed to ZIKV (almost the double) when compared to the downregulated genes (Table 1), but that this upregulation was more intense, with up to 10-fold enhanced expression (S5 Fig). Altogether, these findings reveal that, although many genes were similarly influenced by ZIKV exposure in both myoblasts and myotubes, clear differences were also seen with specific changes in either myoblasts or myotubes.

**Fig 3:**
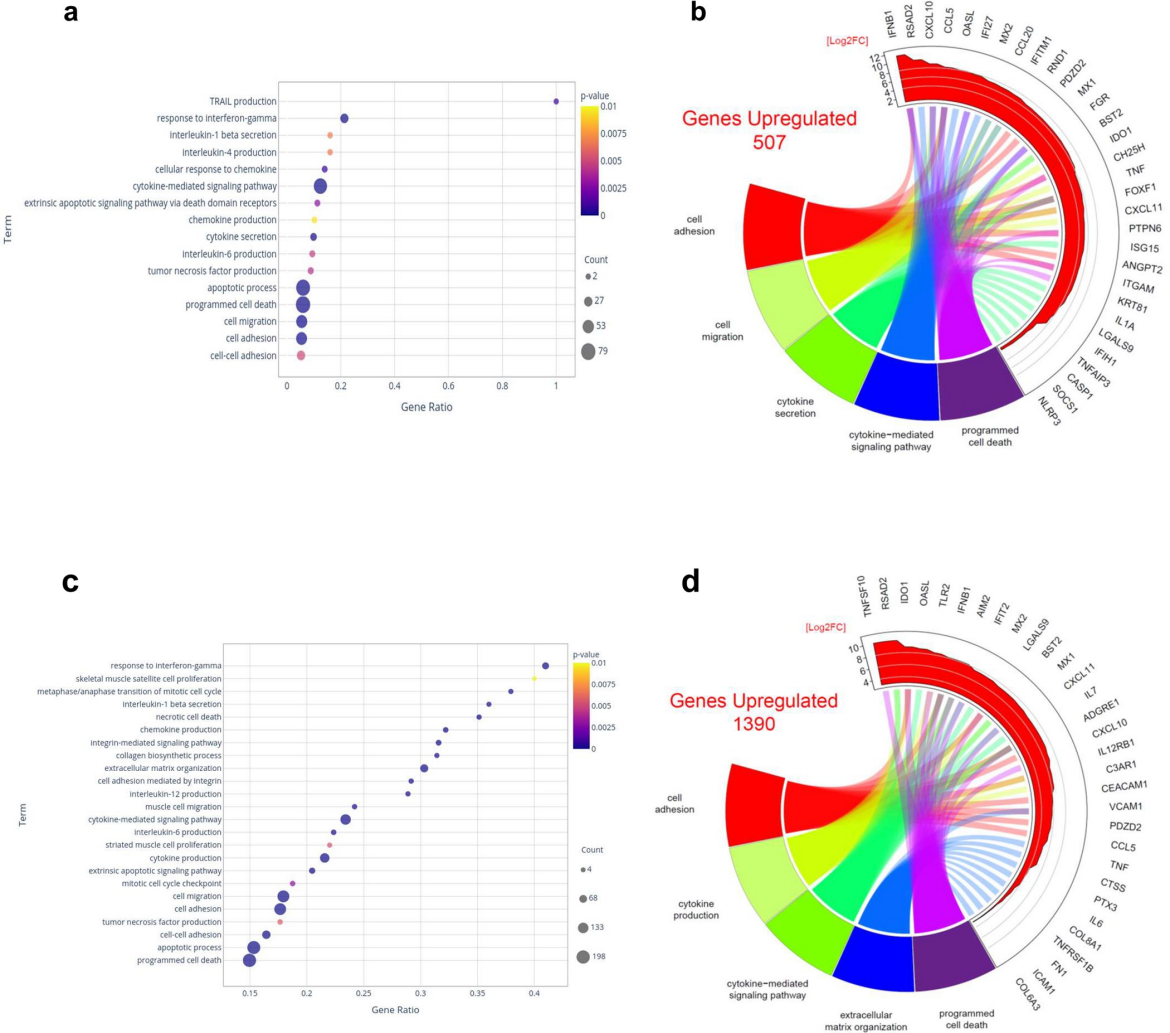
*In vitro* Zika virus infection of human myoblasts and myotubes upregulates gene expression related to cytokine production and signaling, as well as cell adhesion, cell migration and cell death. (**A**) Dot plot illustrating the GO biological process annotations upregulated in myoblasts infected with ZIKV. (**B**) Circos plot representing the total numbers of genes upregulated in ZIKV-infected myoblasts and the 10 most highly upregulated genes among 5 selected GO biological processes. (**C**) Dot plot illustrating the GO biological process annotations upregulated in ZIKV-infected myotubes. (**D**) Circos plot representing the total number of genes upregulated in ZIKV-infected myotubes and the 10 most highly upregulated genes among 5 selected GO biological processes. The whole list of differentially expressed genes can be seen in S1 Table.

Following the general analysis of gene modulation in both ZIKV-treated myoblasts and myotubes, we evaluated the biological pathways significantly enriched after the contact with the virus, applying for that GO, Reactome and KEGG databases (data available at http://biotools.labinfo.lncc.br/muscle_zika). First, we analyzed the pathways related to the muscle system/physiology. As expected, GO analysis of myoblasts revealed an enhancement of pathways related to cell death/apoptosis and cytokine/chemokine production after ZIKV infection. Curiously, pathways related to adhesion and migration were also enhanced by the presence of the virus (Fig 3A). We then chose 5 GO pathways from Fig 3A to highlight the highly expressed genes. Most of them are related to the intrinsic antiviral response, as well as cytokines, adhesion, and apoptotic pathways (Fig 3B).

In ZIKV-treated myotubes, cytokine and chemokine production, extracellular matrix organization and adhesion pathways were enhanced, as was the case for GOs related to migration, proliferation, and cell cycle (Fig 3C). As observed for infected myoblasts, upregulation of several genes from the intrinsic antiviral response, but also genes related to ECM, adhesion, cytokines/chemokines were detected, as seen in the circus plot comprising the 5 chosen GO pathways (Fig 3D). Of note, we found upregulation in the cell death/apoptosis pathways, despite the absence of signs of death or apoptosis.

**Fig 4:**
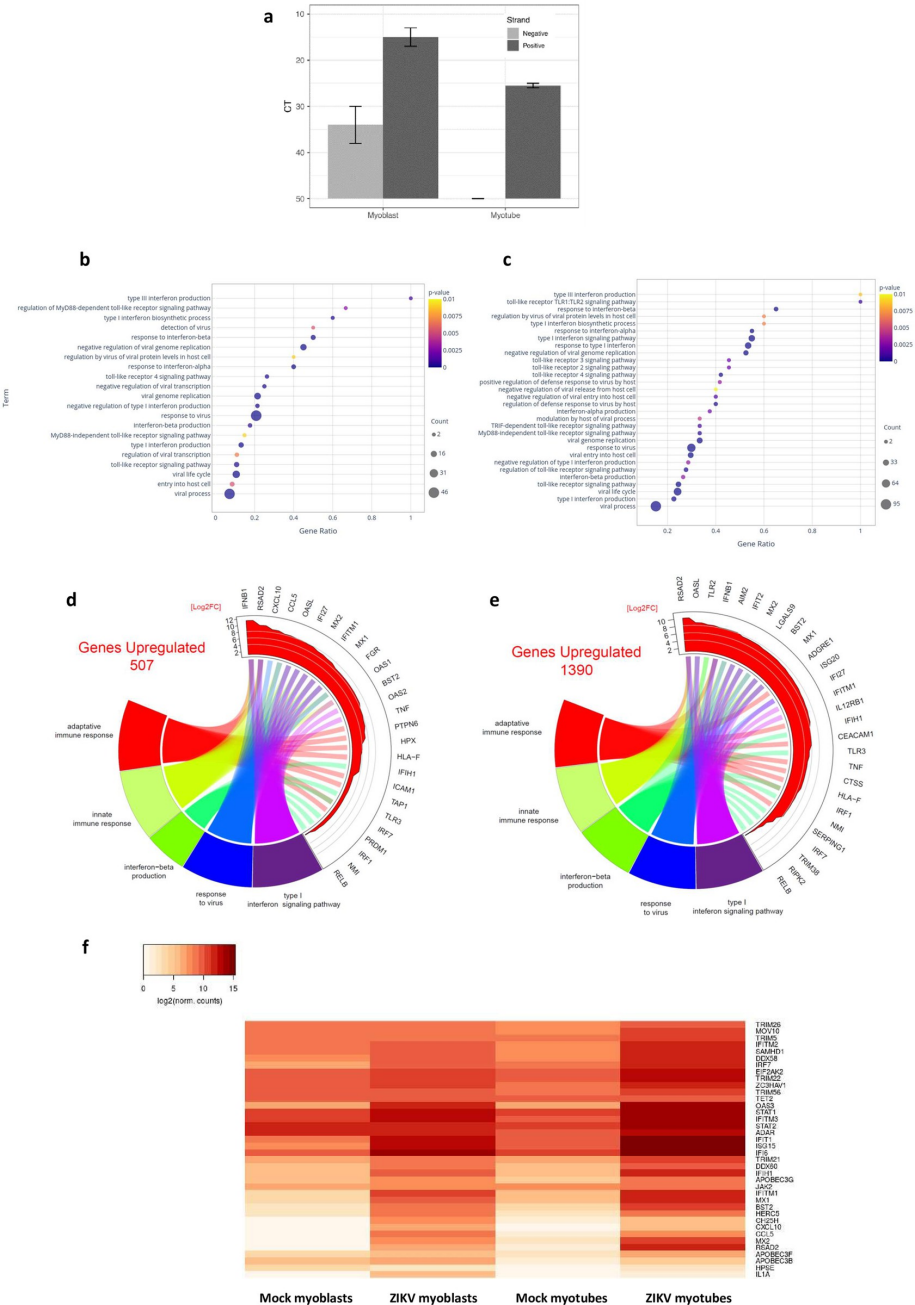
Myoblasts infected with ZIKV and myotubes in contact with ZIKV enhance gene expression related to response to viral infection, as well as innate and adaptive immune responses. (**A**) PCR analysis of positive and negative ZIKV RNA strands in myoblasts (expressing both positive and negative RNA strands) and myotubes (expressing only the positive strand). Gene ontology biological process annotations related to response to viral infection and innate and adaptive immune responses upregulated in myoblasts (**B**) and myotubes (**C**) infected with ZIKV. Circos plot showing the 10 most upregulated genes within each of the 5 selected pathways related to the intrinsic antiviral response (interferon-beta production, response to virus and type I interferon signaling pathway), and innate and adaptive immune responses after ZIKV infection in human myoblasts (**D**) and myotubes (**E**). (**F**) Heatmap of differentially expressed antiviral genes in mock or ZIKV-infected myoblasts and myotubes. The whole list of differentially expressed genes can be seen in S1 Table.

The alterations observed in myotubes could be a consequence of the interaction of the virus with membrane receptors on the cells, possibly stimulating them without internalization of viral particles; alternatively, the virus could enter the cells but not (or barely) replicate. We thus analyzed the expression of both positive and negative strands of the ZIKV RNA in myoblasts and myotubes. We did find the positive, but not the negative, ZIKV RNA sequence within myotubes, strongly suggesting that the virus enters in myotubes but does not progress to RNA replication and protein synthesis, both necessary for productive infection (Fig 4A). As expected, myoblasts presented both positive and negative strands reinforcing the competent virus replication on those cells.

### ZIKV induces the upregulation of genes related to type I interferon, as well as innate and adaptive immune responses

When we analyzed the most upregulated genes in ZIKV-treated myoblasts and myotubes (Fig 3B and 3D, respectively), we noticed that many were part of the immune and antiviral responses.

After defining that ZIKV can enter in myotubes but does not replicate within these cells, we searched for upregulated pathways related to the intrinsic antiviral responses. We observed that both cell types upregulate several GO pathways involved in the antiviral response (Fig 4C and 4D). However, ZIKV-treated myotubes (Fig 4C) displayed antiviral pathways that were not found in infected myoblasts (Fig 4B). Also, most pathways regulated in both cell types presented an increased Gene Ratio in myotubes compared to the same pathways in myoblasts.

We also analyzed antiviral pathways in the Reactome database. Like the GO, a larger number of pathways, some of them including a high number of genes, were upregulated in myotubes compared to myoblasts (S6A and S6B Fig). Of particular interest was the identification of the STING pathway—essential for the antiviral response—that was only upregulated in ZIKV-treated myotubes.

In both myoblasts and myotubes, several GO and Reactome pathways related to the innate and adaptive immune responses were also upregulated, even when we restricted the evaluation to intrinsic antiviral pathways (Figs 7 and S6). As seen in Fig 4, the highest upregulated genes within 5 selected GOs were related to innate and adaptive immune responses as well as to the antiviral response, in both ZIKV-treated myoblasts and myotubes, compared to non-infected cells. Some of those genes were upregulated in myotubes but not in myoblasts, such as TLR2, AIM2, CEACAM1, IL12RB1, TRIM38, RIPK2, IFIT2, LGALS9, ADGRE1, ISG20, CTSS and SERPING1 (Fig 4E).

This further prompted us to investigate the genes involved in mechanisms that could explain myoblast susceptibility and/or myotube resistance to ZIKV productive infection. In this respect, we observed that several genes involved in the inhibition or the control of ZIKV replication were highly upregulated in myotubes, being slightly or not upregulated in infected myoblasts (Fig 4F). Among those genes, some such as the TRIM family (TRIM5, TRIM21, TRIM22, TRIM26 and TRIM56) are involved in the inhibition of virus uncoating and disassembly [21]; while others are related to the inhibition of viral replication, such as ADAR, APOBEC3B, APOBEC3F, APOBEC3G, OAS3, MOV10, SAMHD1 and STING [22–25]. Moreover, we found genes involved in the inhibition of translation and assembly of viral proteins such as the STING, EIF2AK2 and DDX60 [23,26,27]; or involved in the inhibition of the release of newly formed viral particles, for example, the BST2 gene [28].

Some upregulated genes in myotubes are related to viral RNA recognition, such as MDA5 and DDX58/RIG1 [29], and to the response induced by type I IFNs, namely JAK2, STAT1 and STAT2). Type I IFNs are the major products involved in the intrinsic cell response to viruses, with IFN-α/β being upregulated [30]. In our experimental model, both myoblasts and myotubes responded to ZIKV by upregulating IFN-β gene expression (with a log2 fold change of 11.31 in myoblasts and 8.11 in myotubes), but not IFN-α. Also, the GO “Type III Interferon production” was upregulated in both myoblasts and myotubes, with the highest gene ratio (Fig 4C and 4D).

In the absence of infection, we observed that IFN-α and IFN-β genes were not modulated during myoblast differentiation to myotubes. However, the analysis of selected GO terms in myotubes showed that the differentiation process induced the upregulation of the pathway “Response to type I Interferon”, which comprises many other genes involved in the cellular antiviral response (data available at http://biotools.labinfo.lncc.br/muscle_zika/). In contrast, GO processes related to virus entry and viral life cycle were downregulated in myotubes, including the “*Entry into host*”, “*Interactions with the host*”, and “*Positive regulation of viral life cycle processes*”. These results suggest that muscle cell differentiation *per se* makes myotubes more resistant to viral infections by upregulating antiviral genes related to the type I interferon response and downregulating a set of genes, which could facilitate viral entry and replication.

### Zika virus modulates miRNA expression in human myoblasts and myotubes

**Fig 5:**
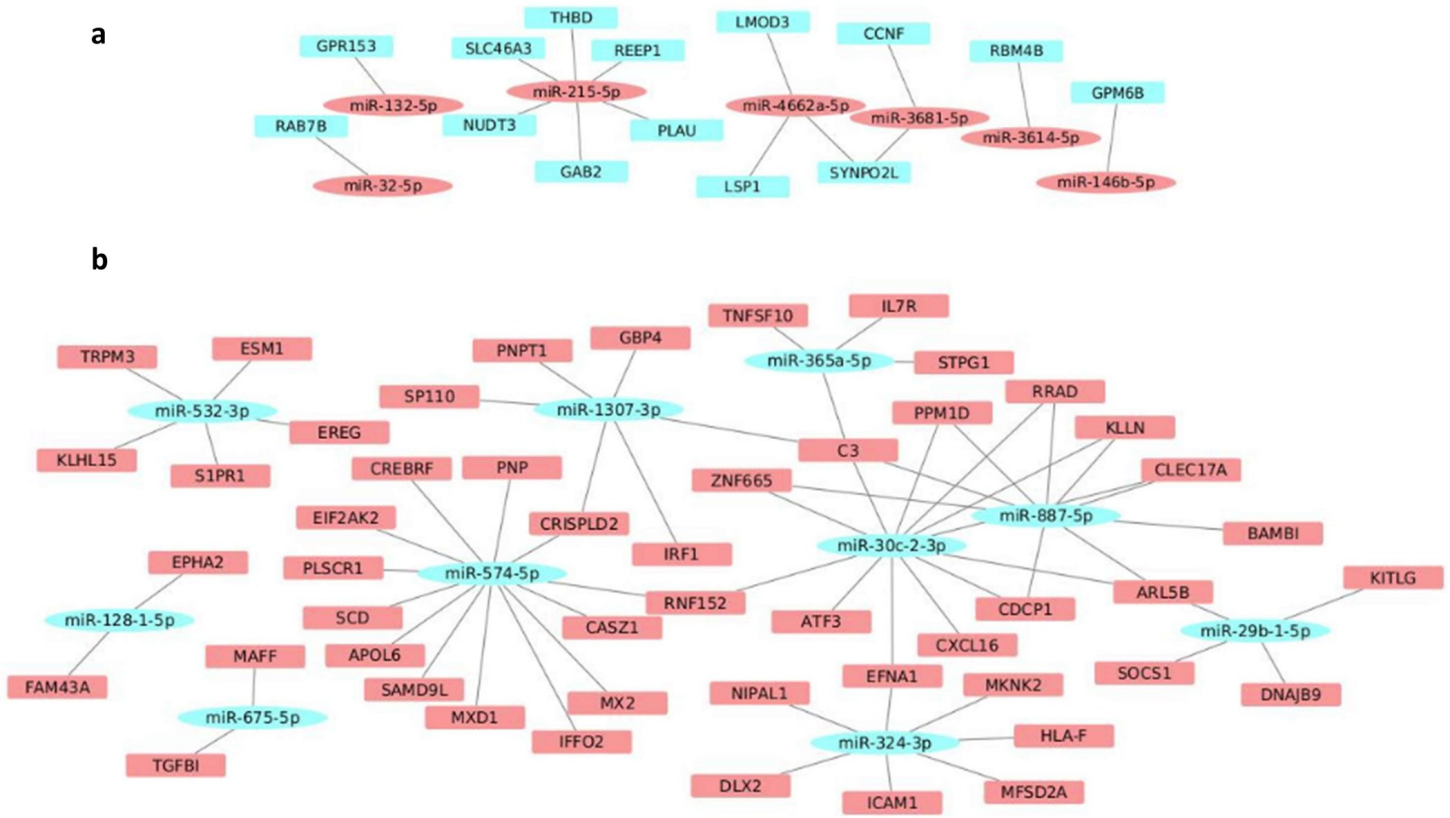
Modulation of miRNAs following *in vitro* ZIKV infection in human myoblasts. (**A**) Network formed by upregulated miRNAs (red ellipses) and their respective targets downregulated (blue rectangles) in ZIKV-infected myoblasts. (**B**) Network formed by downregulated miRNAs (blue ellipses) and their respective targets downregulated (red rectangles) in ZIKV-infected myoblasts. The whole list of differentially expressed miRNAs can be seen in S2 Table.

**Fig 6:**
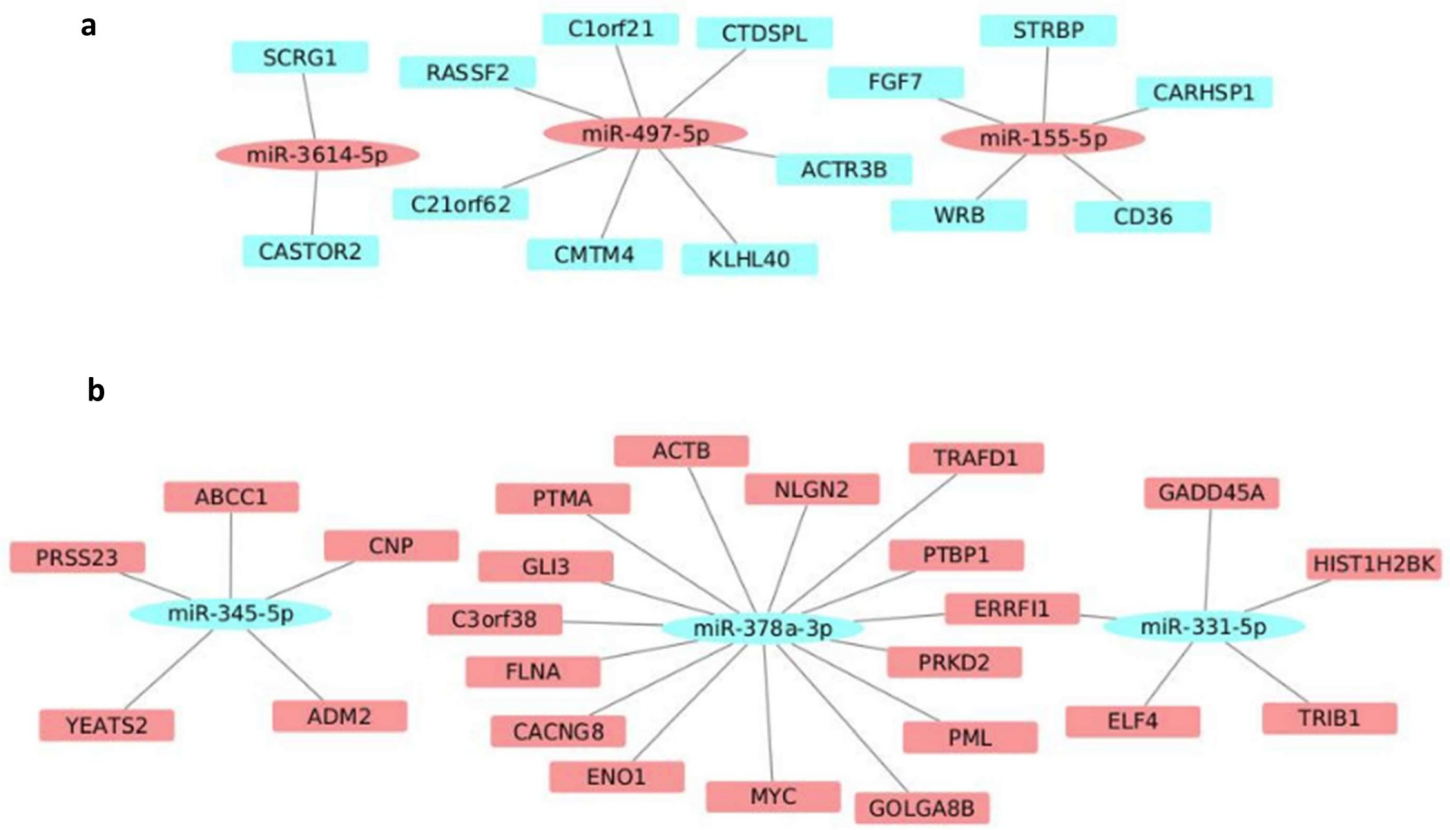
Modulation of miRNAs following *in vitro* ZIKV infection in human myotubes. (**A**) Network formed by upregulated miRNAs (red ellipses) and their respective targets downregulated (blue rectangles) in myotubes exposed to ZIKV. (**B**) Network formed by downregulated miRNAs (blue ellipses) and their respective targets downregulated (red rectangles) in myotubes exposed to ZIKV. The whole list of differentially expressed miRNAs can be seen in S2 Table.

In contrast to the gene expression, ZIKV altered three times more the expression of miRNAs in myoblasts as compared to myotubes (Table 1), with the miR-3614-5p being the most upregulated miRNA in both myoblasts and myotubes with a higher fold change value in the myotubes exposed to ZIKV (S2C and S2D Table). The miRNA-target network of the ZIKV-infected myoblasts presented 7 miRNAs upregulated with 14 downregulated target genes and 10 miRNAs downregulated with 50 upregulated targets genes (Fig 5A and 5B and S2C and S2E Table). The miRNA-target network of ZIKV-treated myotubes revealed 3 upregulated miRNAs with 14 downregulated targets and 3 downregulated miRNAs with 24 upregulated targets (Fig 6A and 6B and S2D and S2F Table).

## Discussion

Our work provided clear evidence that *in vitro* ZIKV exposure to both myoblasts and myotubes results in profound changes in gene expression profiles, comprising both protein-coding genes and microRNAs. Most strikingly were the extensive significant changes seen in myotubes that occurred in the absence of a productive intracellular viral particle replication.

The transformation of myoblasts into myotubes is a complex process that profoundly modifies the skeletal muscle progenitor cell, resulting in cell fusion and syncytium formation, with highly specialized functions, ranging from sarcomeric structure, responsible for muscle contraction, to metabolism [31]. Considering this concept, we first compared the gene expression profiling of “myotubes versus myoblasts” without any infection. The analysis of the 4,500 DEGs, which follows the myoblast differentiation into myotubes, pointed to cell adhesion, extracellular matrix organization, migration, proliferation/cell cycle as the major downregulated pathways. Interestingly, the most downregulated gene was the COL8A1, which encodes the collagen type VIII α1 chain. This protein is produced by satellite cells, promoting their proliferation through the PI3K/AKT signaling pathway [32]. Also significantly downregulated were the CENPF (centromere protein F) gene, which encodes a protein component of the nuclear matrix during the G2 phase of interphase [33], and the HAS2 (hyaluronan synthase 2) gene, which is related to wound healing and cell migration [34,35]. Of note, hyaluronic acid inhibits myoblast fusion and differentiation [36,37]. Other noticeable downregulated genes comprised those encoding the Ki-67 protein and the topoisomerase IIa, well-known cell cycle-related markers [38], the ID1 myogenesis inhibitor [39], and the c-Myb, which inhibit myogenic differentiation through the repression of MyoD [40]. In summary, the functions of these significantly downregulated genes are related to the inhibition of the cell cycle, which is required for muscle differentiation in physiological conditions [41].

In myotubes, the most upregulated GO pathways, compared to myoblasts, were related to skeletal muscle fiber organization, fiber contraction and muscle metabolism. We found that MYBPB1, ACTA1, MYH8, CSRP3, TNNI2, MB, TNNC2, TMOD1, ATPA2A1 and SCN4 are among the most upregulated genes; and all of them being related to muscle integrity, sarcomere structure and contraction, as well as metabolism [42–50]. Corroborating previous findings [51], our results highlight the genes and pathways modulated during the transition of myoblasts to myotubes in non-infective conditions.

A less expected change in gene expression is the upregulation of antiviral pathways, such as “*Response to type I Interferon*”, detected in myotubes compared to myoblasts without any infection. This contrasted with the downregulation of GO processes that facilitate viral life cycle. For example, the AXL gene, coding for one of the ZIKV receptors in different cell types, is highly downregulated in myotubes (data available at http://biotools.labinfo.lncc.br/muscle_zika/). In this sense, the myoblast-into-myotube differentiation process seems to render myotubes more resistant to viral infections when compared to the proliferating muscle progenitor cells. Indeed, our study confirms recent findings showing that human myoblasts are susceptible to ZIKV infection, with the detection of intracellular virus protein and both positive and negative virus RNA strands. Conversely, myotubes are resistant to infection and negative for ZIKV proteins (Legros et al., 2020) [10]. We demonstrated that only the positive virus RNA strands were detected in these cells, showing that despite the downregulation of AXL, ZIKV can infect myotubes but are unable to progress towards a productive transcription and translation to form new virions.

The differentiation-dependent myoblast susceptibility to ZIKV was also observed for other viruses such as CHIKV and Sleeping Disease Virus (SDV) [18,52]. However, this does not seem to be a typical characteristic of skeletal muscle cells since other viruses like dengue, sinbis and influenza also infect myotubes [53–55].

When myoblasts were infected with ZIKV, they underwent important gene modulation, involving significant changes in 713 genes, representing 2.2% of the total DEGs. Although myotubes were resistant to productive infection, the changes in their transcriptome were even more intense than those seen in infected myoblasts, with more than twice as many dysregulated genes. This higher gene modulation may reflect the entry of the virus in myotubes but also the capacity of myotubes to control ZIKV replication.

Expression of interferon type I cytokines is a hallmark of the antiviral host response [30]. Accordingly, both infected myoblasts and myotubes massively upregulated IFN-β gene expression, but not IFN-α, and pathways related to the intrinsic antiviral immune response (particularly those related to the interferon type I pathways). Also, both cell types exposed to ZIKV upregulated several genes and pathways involved in the attraction, activation as well as regulation of cells and molecules from the innate and adaptive immune response. We found several pathways related to the host antiviral response being upregulated in muscle cells three days after infection using GO and Reactome databases. Upon ZIKV exposure, most of these pathways were upregulated in both myoblasts and myotubes, but most of them involved more genes in myotubes. Moreover, some pathways were detected only in myotubes, like the “STING mediated induction of host immune responses”. STING-cGAS signaling leads to the production of several cytokines, including type I interferons [23]. The transcriptome analysis showed that myotubes, resistant to ZIKV infection, expressed more upregulated genes and pathways involved in the antiviral response.

The signaling pathways “Toll-like”, “RIG-I-like”, and “NOD-like” are involved in the initial sensing of viral infection [56]. KEGG analysis revealed that these three pathways were upregulated in both infected myoblasts and myotubes, being more enriched in myotubes than myoblasts (data available at http://biotools.labinfo.lncc.br/muscle_zika). Other important initial sensors for the virus presence are the inflammasomes [57–59]. Despite no significant gene modulation of IL1-β, IL-18 and IL-33, other genes of the inflammasome complex were highly upregulated and again more intensely in myotubes than myoblasts, such as NLRP3 and CASP1, or AIM2; the latter being upregulated only in myotubes.

**Fig 7:**
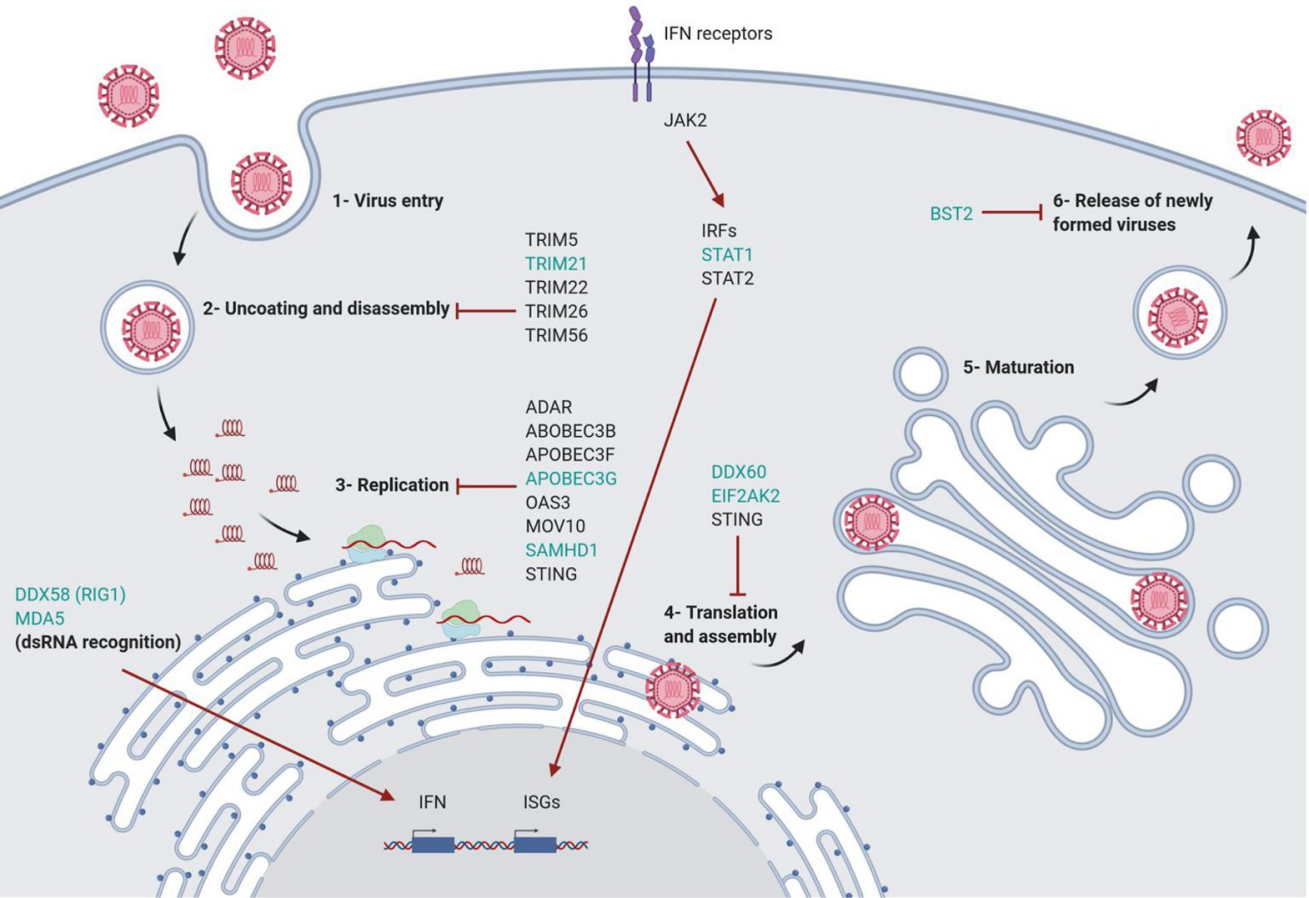
Genes upregulated following *in vitro* ZIKV infection in human myotubes and their role in antiviral responses. Cellular antiviral genes affecting different steps of virus replication were upregulated after ZIKV infection in myotubes but not in myoblasts (genes in black) or that were highly upregulated in myotubes (genes in green) when compared to myoblasts following ZIKV exposure. The ISG15, TRIM5, TRIM21, TRIM22, TRIM26 and TRIM56 genes are involved in the inhibition of virus uncoating and disassembly; ADAR, APOBEC3B, APOBEC3F, APOBEC3G, OAS3, MOV10, SAMHD1 and STING are related to the inhibition of viral replication; EIF2AK2, DDX60 and STING are inhibitors of translation and assembly of viral proteins; BST2 inhibits the release of new viral particles. IFIH1/MDA5 and DDX58/RIG1 recognize viral double-stranded (ds) RNA and transcriptionally induce type 1 IFN, which is subsequently secreted; activation of JAK2, IRFs, and STATs 1 and 2 leads to the expression of interferon-stimulated genes (ISGs), which have different antiviral effects.

Overall, by comparing gene expression changes in both cell types, we showed that different groups of genes related to viral resistance or response were upregulated in ZIKV-exposed myotubes but not (or less upregulated) in infected myoblasts (see Fig 7). These genes included ISG15 and the TRIM family (TRIM5, TRIM21, TRIM22, TRIM26 and TRIM56), which inhibit the virus uncoating and disassembly [21]. Furthermore, genes related to the inhibition of viral replication were upregulated in myotubes, such as ADAR, APOBEC3B, APOBEC3F, APOBEC3G, OAS3, MOV10, SAMHD1, as well as STING, which is involved in the inhibition of translation and assembly of viral proteins, and the EIF2AK2 and DDX60 genes [23–26,60]. The BST2 gene is also upregulated in infected myotubes, which is in keeping with the data showing that the release of new viral particles can be inhibited by BST2 [24].

Other genes upregulated in myotubes are related to virus recognition, such as IFIH1/MDA5 and DDX58/RIG1. They recognize viral double-stranded (ds) RNA and initiate the type 1 IFN transcription pathway, which is involved in the inhibition of ZIKV infection [29]. In parallel, the expression of JAK2, STAT-1 and STAT-2 genes was also upregulated in myotubes. Both are part of type 1 IFNs pathway that induce the transcription of IFN-stimulated genes (ISGs), such as MX1, MX2 and ISG15, among others, that can inhibit viral translation, assembly, uncoating and disassembly, respectively [24]. Overall, these results show that, although we could not detect ZIKV protein in myotubes exposed to the virus, it did induce a significant change in the gene expression profiles in these cells, even more pronounced than those seen in myoblasts. Moreover, many of the highly upregulated genes in ZIKV-treated myotubes were related to the intrinsic response against viruses.

Conceptually, the comparative analysis between the two types of muscle cells highlights pathways and genes that may be responsible for the resistance to ZIKV infection of differentiated muscle cells. These genes or pathways can be modulated in muscle cells prior to infection. Such strategies can indicate the targets that promote ZIKV resistance or susceptibility in the skeletal muscle system and other tissues, such as the CNS, unravelling the biology of ZIKV infection and opening the way to new antiviral drugs.

The skeletal muscle system is not only responsible for maintaining posture and movement. Still, it is also a highly metabolic and endocrine organ that produces soluble factors, such as myokines, which interact with several other organs [61,62]. For example, the prototype myokine IL-6 causes hyperinsulinemia, liver inflammation and other systemic alterations [63]. We observed that the gene encoding this myokine is upregulated in infected myotubes, and the enhancement of IL-6 secretion by skeletal muscle could have impact on the central nervous system, since IL-6 can act synergistically with IL-17 to protect cells from apoptosis, promoting viral persistence [64].

Furthermore, we observed that pathways related to cytokine and chemokine production, adhesion and migration were upregulated, whereas muscle development, filament sliding, and contraction were downregulated in both cell types after ZIKV exposure. Interestingly, GO pathways of cell migration and cell proliferation were upregulated in infected myotubes, suggesting that ZIKV modulates biological processes that should be turned off in muscle cells.

We also evaluated the microRNA expression profile under ZIKV exposure. We found that most miRNAs targets differentially expressed in the myotubes, compared with myoblasts, are related to cell division, chromosome segregation and DNA replication (S3A Fig). Also, the miRNA-target network of the ZIKV-infected myoblasts showed several miRNAs that are likely related to the response to interferon-γ as miR-32-5p (RAB7B), miR-1307-3p (GBP4 and IRF1), miR-324-3p (ICAM1 and HLA-F), miR29b-1-5p (SOCS1) and miR-30c-2-3p (CXCL16).

The miRNAs 141-5p, 370-5p, 29b-5p, 532-3p, which presented changes in expression in ZIKV-infected myoblasts, have also been reported in other viral infections [65]. One of the validated targets of the miR-29b-5p, which is downregulated in ZIKV-infected myoblasts, is the SOCS1 gene. The SOCS (suppressors of cytokine signalling) gene family has been reported to be upregulated in viral infections, promoting the progression of the viral life cycle by inhibiting antiviral signalling pathways and ubiquitination of viral proteins [66].

Despite the limitations of our study, such as the use of an in vitro model with only one cell lineage, one MOI, at one kinetics point, our work reveals that human skeletal muscle cells are targets for ZIKV (even in the absence of virus replication in myotubes) and that these cells undergo a profound modulation of gene expression, including protein-coding genes and miRNAs related to various cell biological responses. The transcriptomic analyses revealing the modulation of genes and miRNAs related to viral resistance and/or response in myotubes point to several pathways potentially involved in the mechanisms of ZIKV resistance or inhibition of productive infection in those differentiated cells and can thus be helpful to define targets for future strategies aiming at ZIKV resistance.

## Methods

### Human skeletal muscle cell cultures

The human LHCN-M2 myoblast cell line was immortalized from satellite cells derived from the pectoralis major muscle of a 41-year-old Caucasian male donor by transduction with retroviral vectors containing the cdk-4 and hTERT genes [19]. We confirmed the myogenicity of the LHCN-M2 myoblasts by the expression of desmin or CD56 ascertained by immunohistochemistry or flow cytometry, respectively. Cultured cells were at least 96% myogenic (see S1 Fig).

Cells were maintained in growth medium composed of DMEM plus medium 199, supplemented with 50μg/ml gentamicin and 20% fetal calf serum (FCS), 25 μg/ml fetuin, 0.5 ng/ml, basic Fibroblast Growth Factor, 5 ng/ml Epidermal Growth Factor and 0.2 μg/ml dexamethasone (proliferation medium). The cells were incubated at 37°C in a 5% CO_2_ atmosphere. Differentiation into myotubes was induced at confluence when the culture medium was switched to DMEM without serum (differentiation medium). After 3 days of differentiation, myotubes were challenged to ZIKV infection.

### Zika virus infection

The ZIKV strain used in this study was the RIO-U1, originally isolated in 2016 from the urine of a patient from Rio de Janeiro, Brazil (GenBank Accession number: KU926309). Viral stocks of ZIKV RIO-U1 were prepared by infecting Vero cell monolayers (ATCC-CCL81). The genome integrity and the infection titer (PFU/ml) were determined as described elsewhere [67].

LHCN-M2 cells in proliferation (named thereafter as myoblasts) and differentiated (the corresponding myotubes) were infected with a 0.1 multiplicity of infection (MOI) of ZIKV diluted in DMEM + 199 medium + 5% FBS or DMEM + 5% FBS respectively, during 2 h. Serum supplementation was set to 5% in both media only during the 2 h incubation with the virus. After incubation, culture media were changed, and LHCN-M2 cells were cultured in proliferation or differentiation media for further 72 hours. Controls (MOCK groups) corresponded to myoblasts or myotubes under the same culture conditions but without ZIKV incubation.

### Flow cytometry

Intracellular staining of viral protein was performed with True-Nuclear transcription factor buffer set (Biolegend, San Diego, USA) as described elsewhere [7]. Briefly, LHCN-M2 cells were incubated with purified pan-flavivirus 4G2 antibody (3 mg/mL–Biomanguinhos, Fiocruz, Brazil) for 1 h at 4°C followed by incubation with fluorescent secondary antibodies (Goat anti-Mouse IgG (H+L) Cross-Adsorbed Secondary Antibody Alexa Fluor 546 # A-11003, or Alexa Fluor 488 # A-11001, ThermoFisher Scientific, MA, USA) for 30 min at 4°C, being then analyzed using a FACS Canto II (BD) flow cytometer, applying the FACS Diva analysis software. We also confirmed the myogenicity of the cells by the expression of CD56 by flow cytometry using an anti-CD56 PE (#555516, BD Biosciences, NJ, USA).

### Immunohistochemistry

LHCN-M2 cells were seeded in 8 well Lab-Tek Chamber Slide System, in a concentration of 10^3^ cells/well for proliferation or 10^5^ cells/well for differentiation conditions, being then infected as described above. After infection, the slides were washed twice with PBS and fixed with 4% paraformaldehyde for 15 min. Slides were treated with permeabilization buffer (0.1% saponin and 1% BSA–bovine albumin serum) for 15 min at 37°C. After that, samples were incubated for 40 min with a blocking solution (2% normal goat serum, 1% FCS, 1% BSA, 0.3% X-100 triton) and submitted to staining with primary antibody for 1 h with the corresponding secondary antibody for 30 min. After staining, the slides were washed twice with a permeabilization buffer, stained with DAPI for 10 min (D9542, SigmaAldrich), washed and mounted with the Dako Fluorescent Mounting Medium (Agilent, Santa Clara, USA). Negative controls, in which the secondary antibody was used alone, did not generate any significant labeling. The percentage of infection was ascertained by counting the numbers of 4G2^+^ cells, divided by the total number of cells per microscopic field. We analyzed 5 fields from 3 independent experiments, and each experiment was performed in triplicate. We confirmed the myogenicity of the LHCN-M2 human myoblasts by monitoring the expression of desmin. For that, cells were fixed using absolute cold ethanol and washed with PBS. Slides were treated with a blocking solution (2% FBS) for 20 min at room temperature. Primary antibodies were incubated for 1 hour in the same conditions and revealed using fluorescent-labeled secondary antibodies. We used anti-Desmin antibody (ab15200 –Abcam, MA, USA) revealed with a Goat anti-Rabbit IgG (H+L) Cross-Adsorbed Secondary Antibody, Alexa Fluor 488 # A-1108, ThermoFisher Scientific), and anti-MyHC antibody (ab124205 - Abcam, MA, USA) revealed with a Goat anti-Rabbit IgG (H+L) Cross-Adsorbed Secondary Antibody, Alexa Fluor 488 # A-1108, ThermoFisher Scientific). Immunostained samples were analyzed using the AxioImager A2 device with the AxioVision Rel 4.8 software (Zeiss, Oberkochen, Germany) or with the Leica TSC SP8 Confocal device with LAS-X Software (Leica Microsystems, Wetzlar, Germany).

### RNA extraction

LHNC-M2 (10^6^) cells were seeded in 150 cm^2^ Petri Plates and 24 h later infected as described previously. Seventy-two hours post-infection, RNA extraction was performed with MOCK and ZIKV-infected myoblasts, using the RNeasy Plus Mini Kit (Qiagen, Hilden, Germany), following the manufacturer’s instructions for mRNA and small RNA extraction. Total RNA concentration and purity were determined by Nanodrop spectrophotometer. RNA integrity was verified through Agilent 2100 Bioanalyzer (Agilent).

### Transcriptome and small RNA library preparation and sequencing

From total RNA, 1 ug was used to prepare 12 small RNA libraries with the TruSeq Small RNA Library Prep Kit (Illumina, USA). The protocol was followed as described by the manufacturer, except for the cDNA construction purification step, which was carried out using 3% Agarose Gel Cassette for targets between 100 bp—250 bp in a BluePippin system (Sage Science, USA). Five hundred ng from the same total RNAs were used to prepare 12 transcriptome libraries with the TruSeq Stranded Total RNA Sample Preparation Kit with Ribo-Zero Gold (Illumina). To check library sizes, we applied the 2100 Bioanalyzer System with the Agilent High Sensitivity DNA Kit (Agilent). Libraries were quantified by qPCR using a KAPA Library Quantification Kit for Illumina platforms (KAPA Biosystems, USA). Sequencing was performed on a NextSeq 500 System (Illumina). For the small RNA libraries, we used a NextSeq 500/550 High Output v2 kit (75 cycles), and 1x50 bp single-end reads were obtained. We used a NextSeq 500/550 High Output v2 kit (150 cycles) for the transcriptome libraries, and 2x75 bp paired-end reads were obtained.

### Transcriptome analysis

The quality of the 12 transcriptomes and 12 small RNA libraries were checked by FASTQC (https://www.bioinformatics.babraham.ac.uk/projects/fastqc/), and the trimming was done using Trimmomatic [68]. After trimming, reads from 16 to 28 nucleotides were kept for posterior analyses in the small RNA libraries and only reads with at least 35 nucleotides were kept in the transcriptome libraries. The STAR tool [69], version 2.6, was applied to map the transcriptome libraries on the Human genome (GRCH38.p12) from ENSEMBL databank (http://www.ensembl.org/info/data/ftp/index.html) using the default parameters. The small RNA libraries were mapped on the microRNA precursors from humans (Release 22 of miRBase) (http://www.mirbase.org) with the Bowtie2 tool [70], version 2.2.1, using the “—very-sensitive-local” and the “—norc” parameters.

The DESeq2 package [71] from R was used for differential gene expression analysis using three biological replicates. Only the protein-coding, long non-coding, and microRNAs with adjusted p-value < 0.01 and Log2 Fold Change > |1.0| were considered for downstream analyses.

The experimentally validated microRNA-target interactions from miRTarBase (http://mirtarbase.mbc.nctu.edu.tw/php/index.php) were used to search for target mRNAs. Only the validated target mRNAs with inverted expression patterns from their miRNAs were considered for further analysis.

### Enrichment analysis of DEGs

The GO tool was used to analyze the enrichment of biological processes [72]. The transcriptome analysis was performed by the R package GOstats, and the analysis of the miRNA targets was performed by R package ClusterProfiler [73]. The Benjamini & Hochberg (BH) adjusted p-value method was applied for the enrichment with a cutoff of 0.01. The R package ReactomePA [74] was used for the enrichment of the pathways with an adjusted p-value cutoff of 0.1.

### RT-qPCR for intracellular ZIKV detection

Intracellular RNA was analyzed by a one-step RT-qPCR for viral negative-strand or total viral RNA with ZIKV specific primers. First, total viral RNA was harvested using QIAamp Viral RNA Mini Kit (Qiagen), according to manufacturer instructions, then RT-qPCR was performed using GoTaq Probe 1-Step RT-qPCR System (Promega) and primers and probes specific for Zika virus envelope gene [75]. To capture RNA negative strand, only forward primer was used, as previously described [76,77]. Cycle threshold data were analyzed and plotted using the R program.

## Supporting information

S1 Fig Immunophenotypic characterization of the human LHCN-M2 myoblast cell line. Panel (**A**) depicts flow cytometry histograms for the expression of CD56 (green plot), as compared with an unrelated isotype matched immunoglobulin (blue plot). In panel (**B**), adhered cells were immunolabeled for the presence of desmin (in green), thus confirming the myogenicity. Cell nuclei are labelled in blue by DAPI. The bars indicate magnification.
(TIF)

S2 Fig Volcano-plot of gene expression profiles, comparing myotubes over myoblasts, in the absence of Zika virus. The red dots represent the genes that are over the cutoff of Log2 Fold Change > [1.0] and adjusted p-value < 0.05.
(TIF)

S3 Fig Differentially expressed miRNA-targets during myoblast differentiation. Cnetplot representing validated miRNA targets that present the inverse expression pattern from the miRNAs in myotubes compared to myoblasts. Grey circles indicate the GO terms (Biological processes), and their size represents the number of genes for each term. Colored dots represent the Log2 Fold Changes. Purplier colours indicate the most upregulated genes, and greener colours indicate the most downregulated genes.
(TIF)

S4 Fig Venn diagram showing the shared and specific genes from both Mock and ZIKV-infected myoblasts and myotubes separated by expression pattern. Only genes with adjusted p-value < 0.05 and Log_2_ Fold Change > [1.0] are presented in this diagram. The total number of genes from each group are shown in parentheses. Genes can be identified at http://biotools.labinfo.lncc.br/muscle_zika.
(TIF)

S5 Fig Volcano-plot of gene expression profile, comparing uninfected (Mock) *versus* ZIKV-treated myoblasts and myotubes. The red dots represent the genes that are over the cutoff of Log2 Fold Change > [1.0] and adjusted p-value < 0.05.
(TIF)

S6 Fig Reactome biological process annotations related to response to viral infection, innate and adaptive immune responses differentially expressed in muscle cells after ZIKV infection. Graphs show biological processes upregulated in human myoblasts (**A**) and myotubes (**B**) following ZIKV infection.
(TIF)

S7 Fig Selected GO biological process annotations differentially expressed in muscle cells after ZIKV infection. Graphs show immune response related biological processes in human myoblasts (**A**) and myotubes (**B**) following ZIKV infection.
(TIF)

S1 Table Differentially expressed genes in muscle cells during differentiation or after ZIKV infection. (**A**) Upregulated genes in mock myotubes over mock myoblasts; (**B**) Downregulated genes in mock myotubes over mock myoblasts; (**C**) Upregulated genes in ZIKV-infected myoblasts over mock myoblasts; (**D**) Downregulated genes in ZIKV-infected myoblasts over mock myoblasts; (**E**) Upregulated genes in ZIKV-infected myotubes over mock myotubes; (**F**) Downregulated genes in ZIKV-infected myotubes over mock myotubes.
(XLSX)

S2 Table Differentially expressed miRNAs in muscle cells during differentiation or after ZIKV infection. (**A**) Differentially expressed miRNAs in mock myotubes over mock myoblasts; (**B**) Differentially expressed miRNAs in mock myotubes over mock myoblasts and their validated targets; (**C**) Differentially expressed miRNAs in ZIKV-infected myoblasts; (**D**) Differentially expressed miRNAs in ZIKV-infected myotubes; (**E**) Differentially expressed miRNAs in ZIKV-infected myoblasts and their validated targets; (**F**) Differentially expressed miRNAs in ZIKV-infected myotubes and their validated targets.
(XLSX)
