# Supplementary figures and images for: Zika virus disrupts gene expression in human myoblasts and myotubes: Relationship with susceptibility to infection
Source: PLoS Negl Trop Dis. 2022 Feb 16;16(2):e0010166. doi: 10.1371/journal.pntd.0010166 (PMC8923442; doi:10.1371/journal.pntd.0010166)

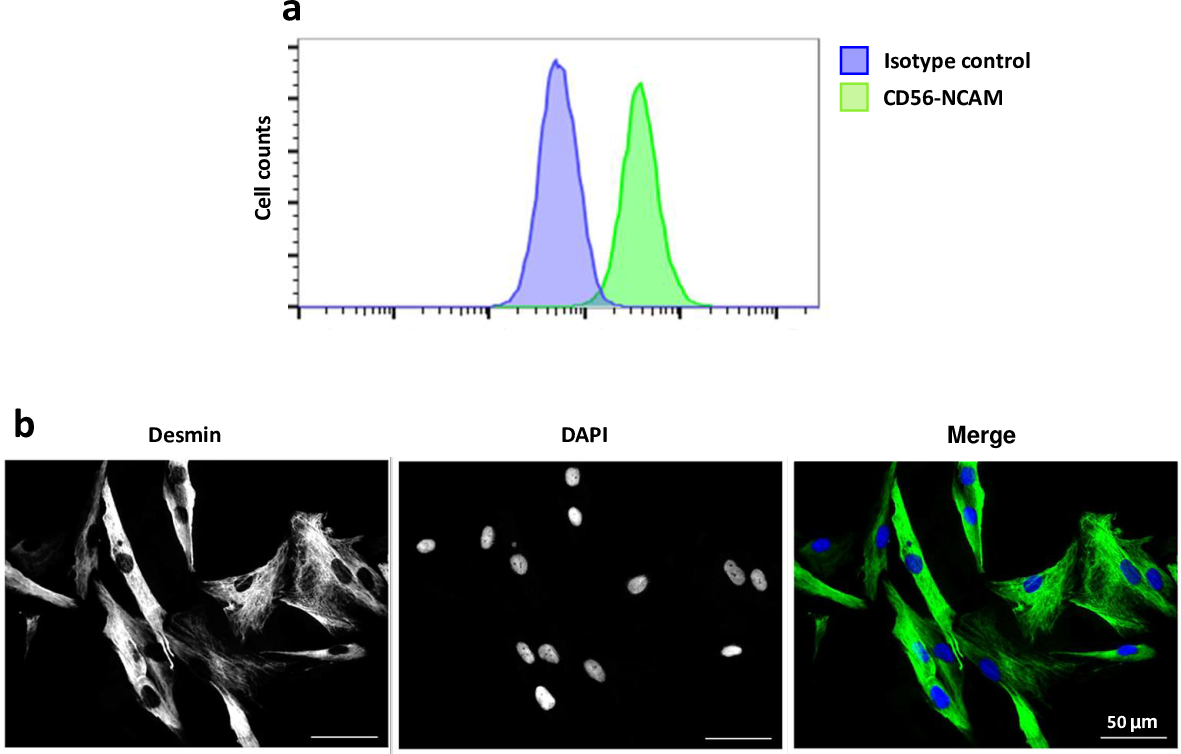

Supplement: S1 Fig — Panel (A) depicts flow cytometry histograms for the expression of CD56 (green plot), as compared with an unrelated isotype matched immunoglobulin (blue plot). In panel (B), adhered cells were immunolabeled for the presence of desmin (in green), thus confirming the myogenicity. Cell nuclei are labelled in blue by DAPI. The bars indicate magnification. (TIF) [file pntd.0010166.s001.tif]

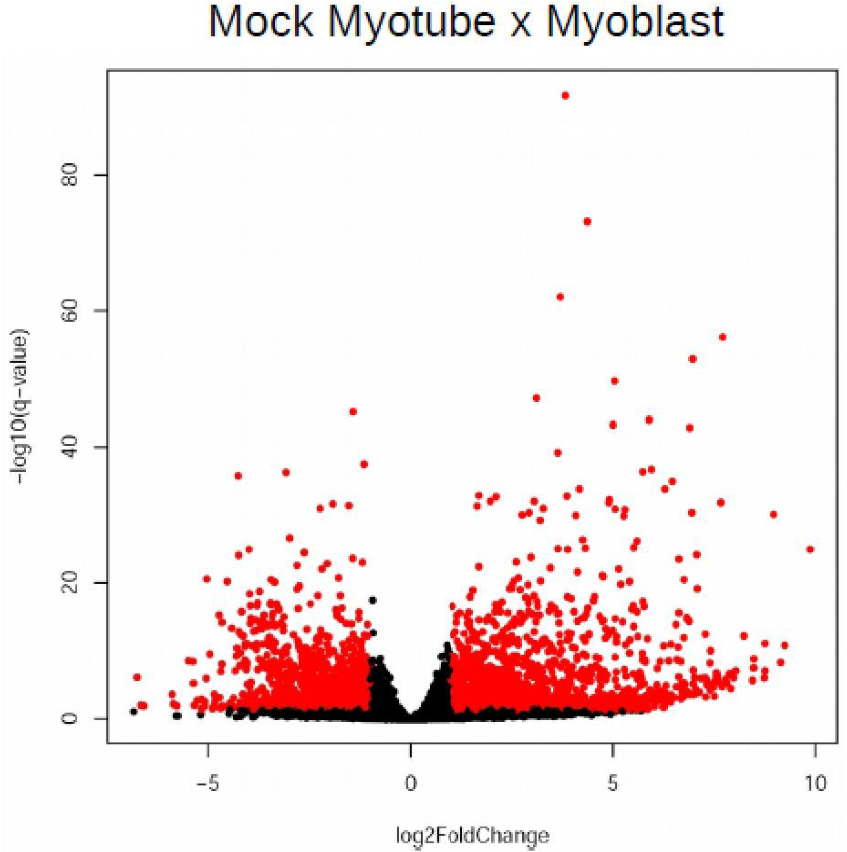

Supplement: S2 Fig — The red dots represent the genes that are over the cutoff of Log2 Fold Change > [1.0] and adjusted p-value < 0.05. (TIF) [file pntd.0010166.s002.tif]

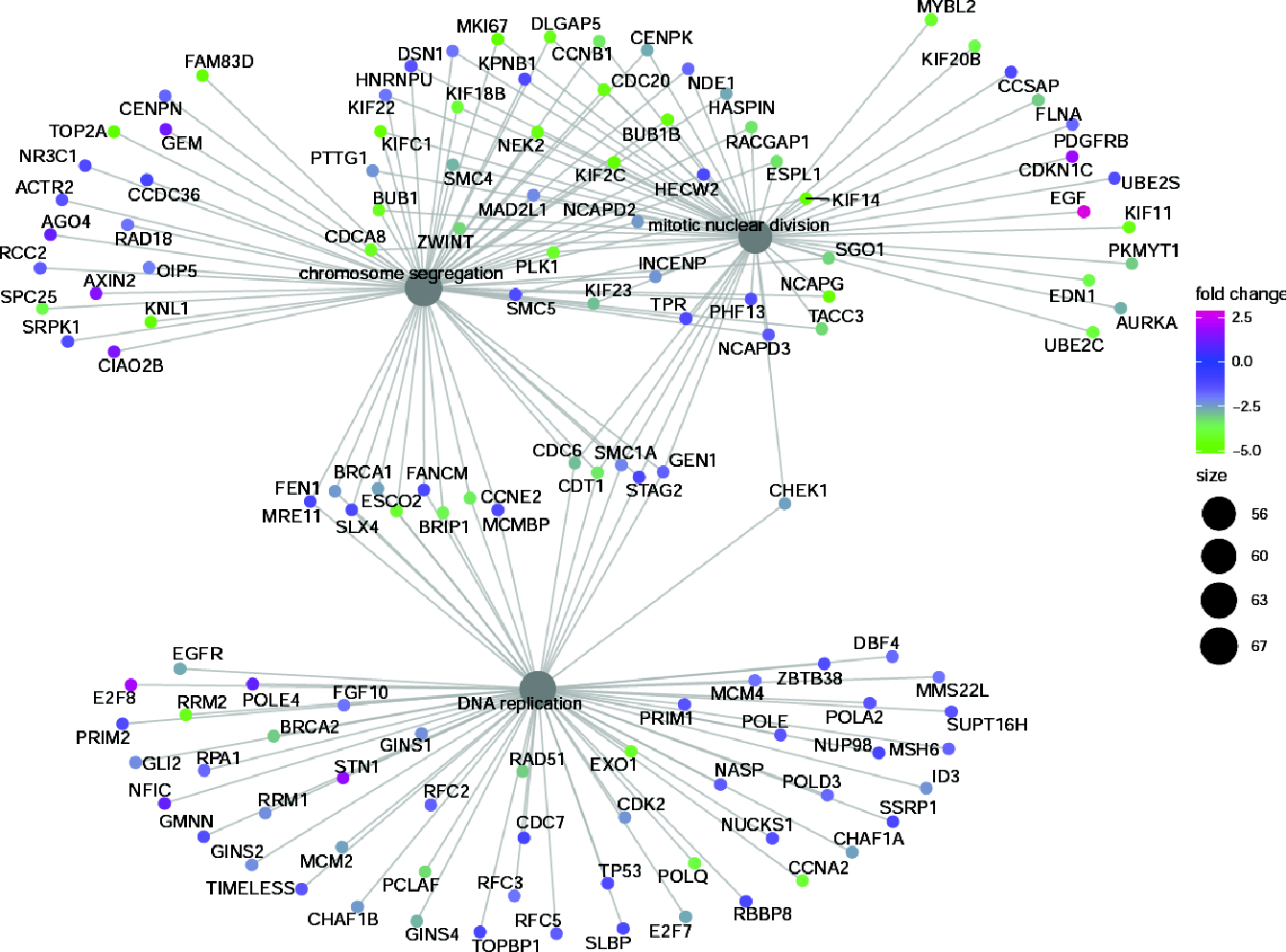

Supplement: S3 Fig — Cnetplot representing validated miRNA targets that present the inverse expression pattern from the miRNAs in myotubes compared to myoblasts. Grey circles indicate the GO terms (Biological processes), and their size represents the number of genes for each term. Colored dots represent the Log2 Fold Changes. Purplier colours indicate the most upregulated genes, and greener colours indicate the most downregulated genes. (TIF) [file pntd.0010166.s003.tif]

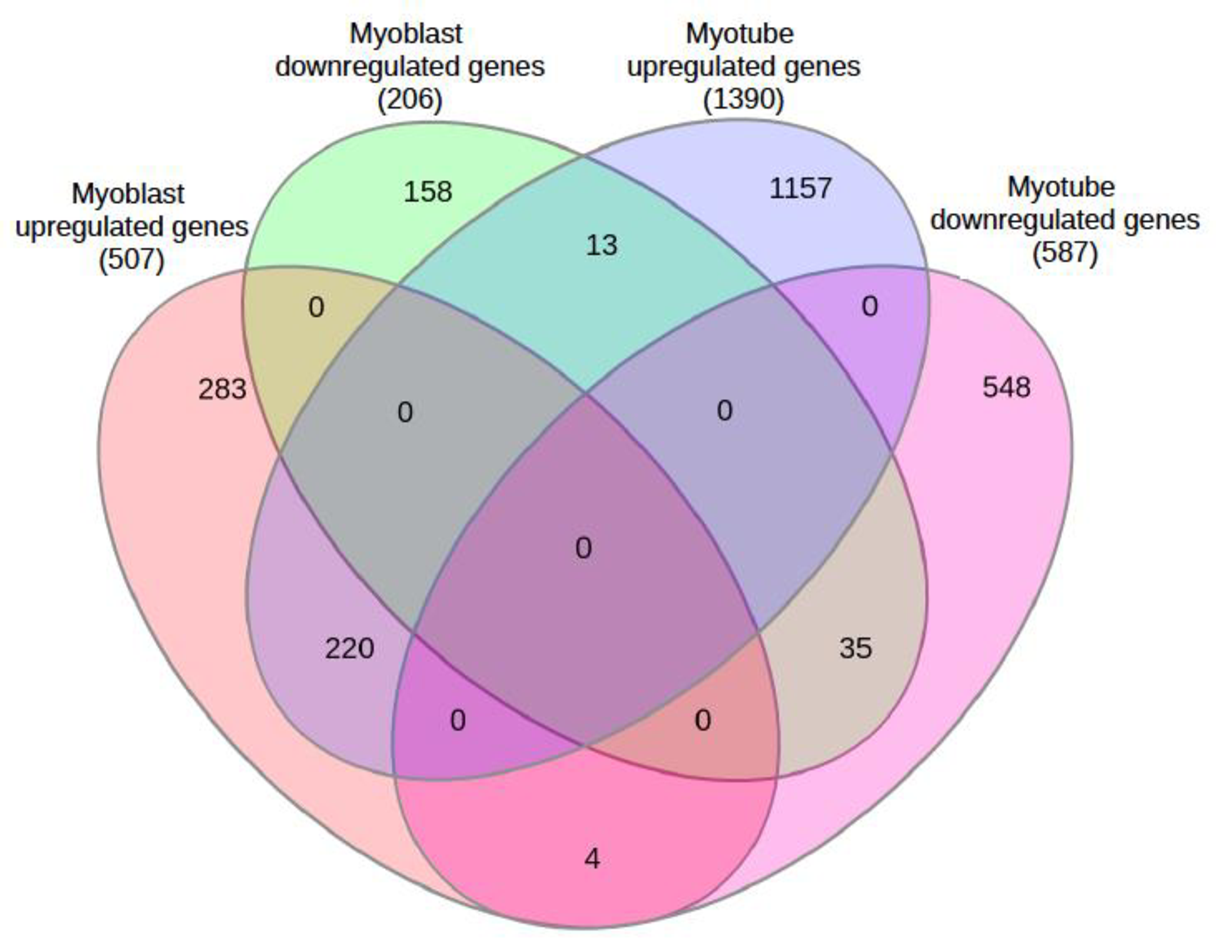

Supplement: S4 Fig — Only genes with adjusted p-value < 0.05 and Log2 Fold Change > [1.0] are presented in this diagram. The total number of genes from each group are shown in parentheses. Genes can be identified at http://biotools.labinfo.lncc.br/muscle_zika. (TIF) [file pntd.0010166.s004.tif]

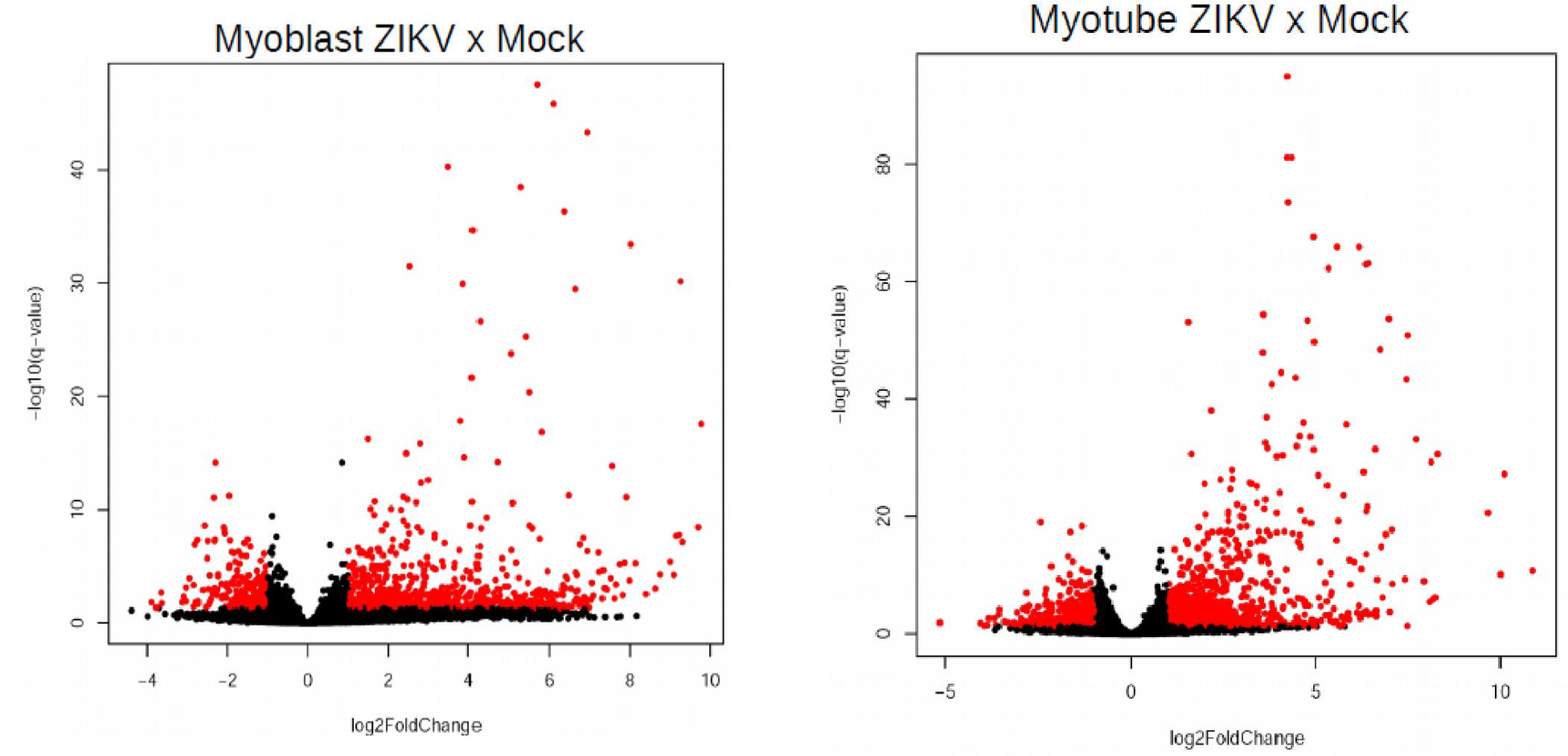

Supplement: S5 Fig — The red dots represent the genes that are over the cutoff of Log2 Fold Change > [1.0] and adjusted p-value < 0.05. (TIF) [file pntd.0010166.s005.tif]

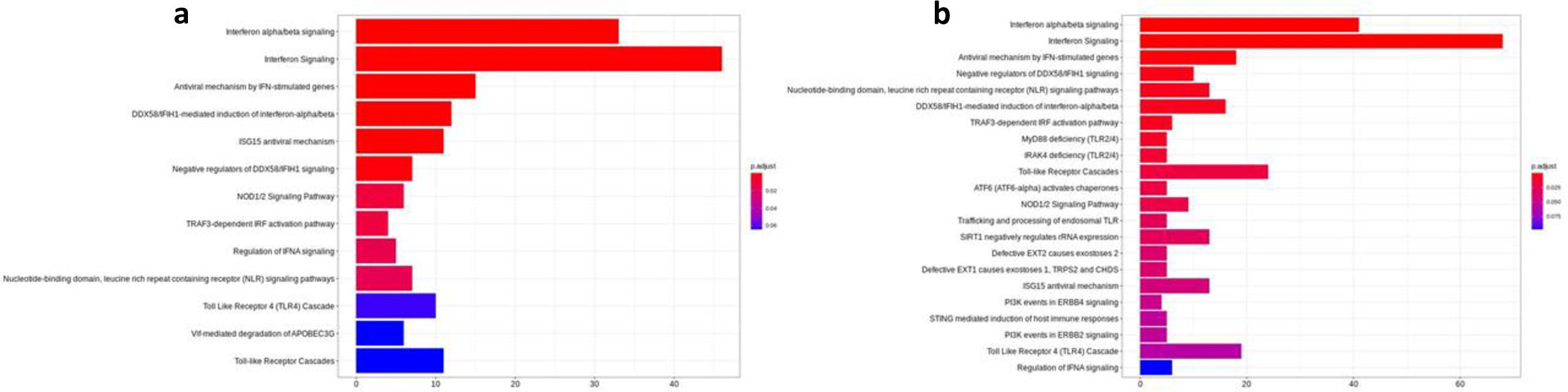

Supplement: S6 Fig — Graphs show biological processes upregulated in human myoblasts (A) and myotubes (B) following ZIKV infection. (TIF) [file pntd.0010166.s006.tif]

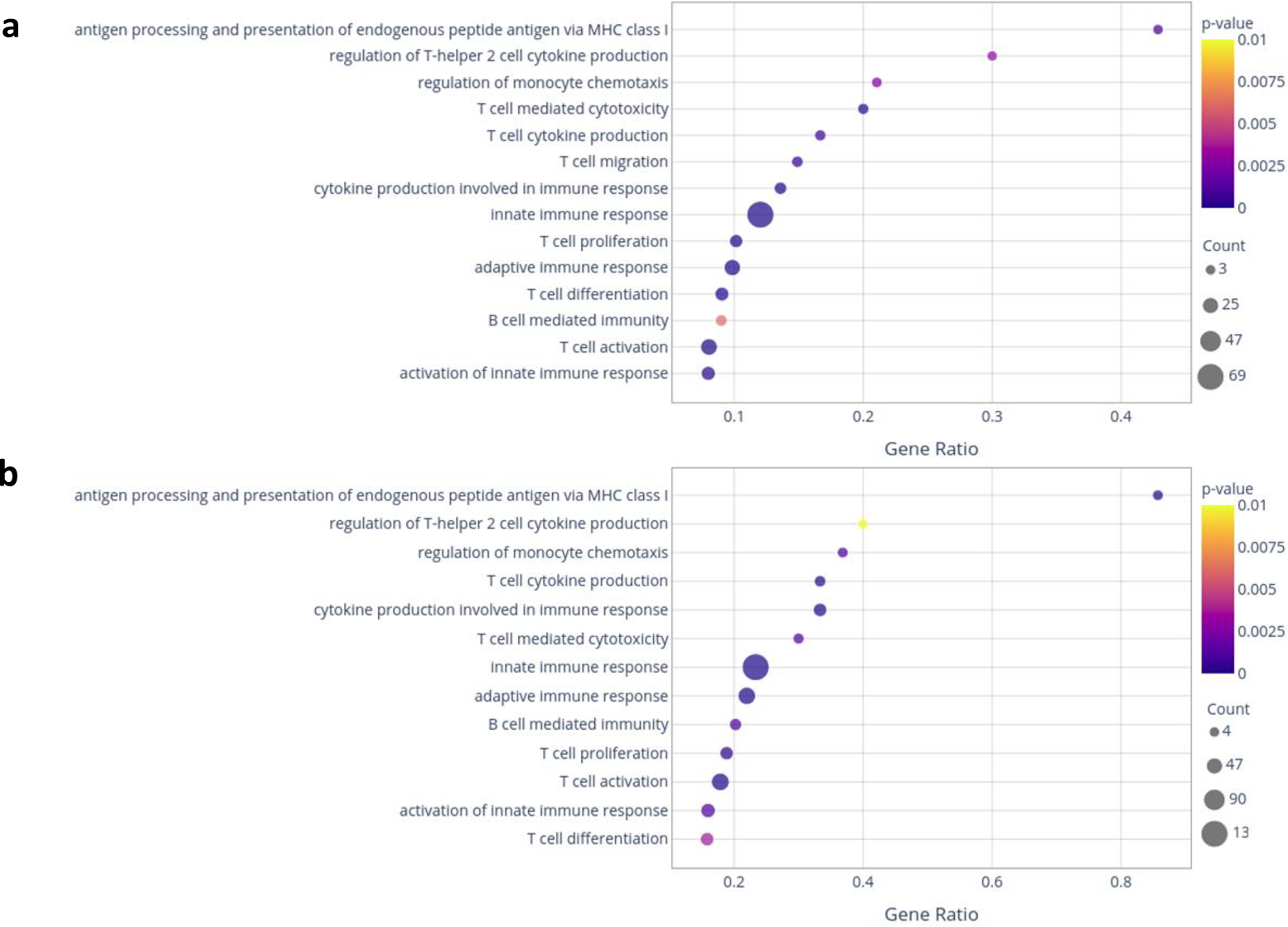

Supplement: S7 Fig — Graphs show immune response related biological processes in human myoblasts (A) and myotubes (B) following ZIKV infection. (TIF) [file pntd.0010166.s007.tif]
